# Variability of release rate of flame retardants in wastewater treatment plants

**DOI:** 10.1007/s11356-018-3403-2

**Published:** 2018-10-15

**Authors:** Jesse Shen, Shirley Anne Smyth, Ronald Droste, Danaëlle Delâge

**Affiliations:** 1 Science and Risk Assessment Directorate, Science and Technology Branch, Environment and Climate Change Canada, 351 Saint Joseph Boulevard, Gatineau, Quebec K1A 0H3 Canada; 2 Department of Civil Engineering, University of Ottawa, Ottawa, ON K1N 6N5 Canada

**Keywords:** Wastewater, Flame retardants, Release variability, Release fraction, Biosolids, Sludge

## Abstract

Information on variability is important in the assessment of the releases and potential risks of brominated flame retardants (BRFs) in the environment, but related data are limited. In this study, two release-characterizing parameters, release fraction to final effluent and influent-biosolids transfer coefficient, were used to quantify releases of five BFRs from eight secondary wastewater treatment plants (WWTPs). The five BFRs are recalcitrant, hydrophobic, and low in volatility. The two parameters for these BFRs were found to vary from day to day and season to season within individual WWTPs as well as from one WWTP to another. These temporal and spatial variations were, however, comparable to each other and both within a factor of 3 above or below the parameter averages. Averages for release fraction were in the range of 0.02–0.29 and those for influent-biosolids transfer coefficient in the range of 3–26 L/g, depending upon a given BFR at a given WWTP. These ranges and the observed factor-3 variability are not only useful for estimating releases of the five BFRs, but more importantly provide read-across data for the assessment of substances with similar physical-chemical properties.

**Electronic supplementary material:**

The online version of this article (10.1007/s11356-018-3403-2) contains supplementary material, which is available to authorized users.

## Introduction

This study addresses data gaps in the release variability of several important brominated flame retardants (BFRs) from wastewater treatment plants (WWTPs). The studied BFRs belong to the family of polybrominated diphenyl ethers (PBDEs) and their replacements. PBDEs have been found to have harmful effects on both the environment (Environment Canada 2006) and human health (Darnerud 2003; US 2009; Wikoff and Birnbaum 2011). In Canada, regulations on PBDEs were enacted in 2008 and their monitoring in various matrices, including wastewater, was put in place thereafter (Government of Canada 2009). Various regulatory actions were also taken in the United States (US) (Ward et al. 2008), the European Union (European Commission 2011), and Asia (Chen et al. 2012). Many novel BFRs have been adopted by industry to replace PBDEs (Renner 2004; Covaci et al. 2011). Despite this worldwide shift, PBDEs still remain a concern because they are present in a wide range of consumer products and continue to be released to the environment via wastewater treatment plants (Andrade et al. 2015). At the same time, certain novel BFRs which have the same fate as PBDEs have been reported as having potential to bioaccumulate in aquatic organisms (Wu et al. 2011) and to persist in the environment (Gouteux et al. 2008). This potential deserves attention in light of an expected increase in use volumes for these novel BFRs.

In Canada, chemical substances including BFRs are assessed for their potential risks to the environment and human health under the Canadian Environmental Protection Act (CEPA) by Environment and Climate Change Canada and Health Canada (ECCC and HC 2016). These assessments focus on determining the exposure and hazard potential of a substance using available empirical and modeled data, including examination of the associated variability and uncertainty of the data. If a substance is determined to pose a risk under CEPA, as in the case of PBDEs, appropriate risk management tools such as regulations, pollution prevention planning notices, guidelines, or codes of practice are introduced to mitigate the risks posed by the substance.

Variability is an important aspect of the occurrences of BFRs in the environment. Their concentrations have been measured in fresh water, sediment, and soil, and showed a large degree of variability (Moon et al. 2012; Wang et al. 2011; Yun et al. 2008; Samara et al. 2006; Petrovic et al. 2004). Such variability presents a particular challenge to the assessment of the effectiveness of control measures for PBDEs as well as to the assessment of the potential risk posed by novel BFRs and other flame retardants. This is due to the fact that the scopes of these assessments often encompass large spatial-temporal scales, while monitoring or measured data are limited to selected times and sites. A major source of this exposure-related variability lies in WWTPs as they are a common entry point for BFRs and many other substances into receiving water via effluent discharge and soil via biosolids land application. A proper quantification for the extent of release variability is, therefore, key in the effort to determine a full range of environmental exposure to a substance.

Despite its importance to the regulatory community, the extent of release variability from WWTPs is not well characterized for the purposes of risk assessment and risk management. Many studies on release variability are limited to ranges of removal rates or concentrations. These include BFR-specific studies focusing on influent and effluent concentrations (Deng et al. 2015; de Boer et al. 2003), seasonal removal variability (Rocha-Gutierrez and Lee 2013), and temporal and geographic distributions in biosolids (Hale et al. 2012). They also include removal variability studies of other substances such as pharmaceuticals (Metcalfe et al. 2003), fragrance substances (Simonich et al. 2002), and polycyclic aromatic hydrocarbons (PAHs) (Manoli and Samara 2008). In addition, there are large influent and effluent concentration datasets reviewed or compiled for substances in personal care products (Hopkins and Blaney 2016) and chlorinated hydrocarbons, PAHs, and metals (Monteith 1987 and Canviro Consultants 1991). However, no practical rule has been derived from these studies for use in release estimation for situations where only limited empirical data on removal rates is available, or model estimates of removal must be used.

This study was a continued effort from the previous work by Kim et al. (2013a, b and 2014) and aimed at addressing exposure-specific data gaps in WWTPs’ release variability. The previous work focused on the determination of concentrations and removal rates of important PBDE congeners and new BFRs in various WWTPs. The present study focused on a new area of direct relevance to release estimation by quantifying release-characterizing parameters and their spatiotemporal variabilities based on the field data previously obtained. The findings from the present study are not only useful for the substances analyzed, but can also apply to those with similar physical-chemical properties.

## Material and methods

### WWTPs

**Fig. 1:**
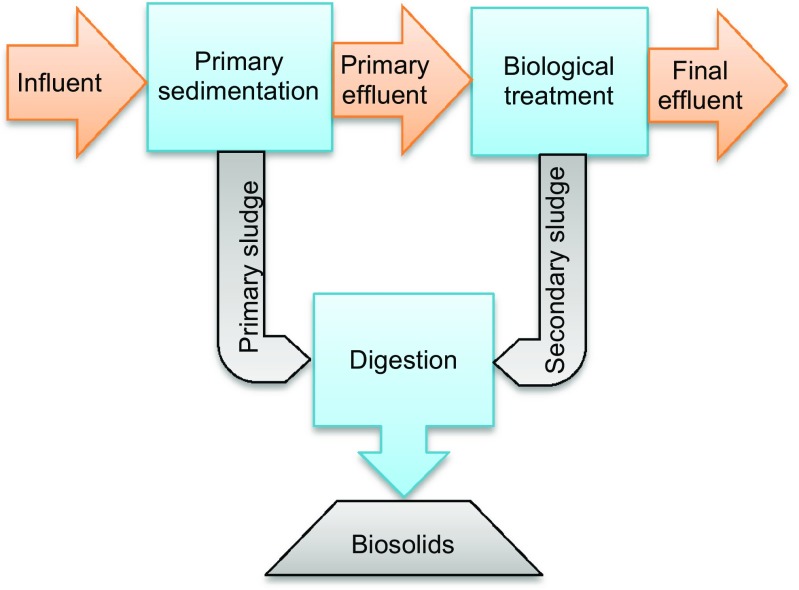
Schematic of wastewater treatment plants selected (Six AS plants, one BAF, and one TF)

Eight Canadian municipal WWTPs were selected in this study, all consisting of primary sedimentation and secondary biological treatment (Fig. 1). The biological treatment includes activated sludge (AS), biological active filter (BAF), and trickling filter (TF). The eight plants are located in five provinces (British Columbia, Manitoba, New Brunswick, Ontario and Prince Edward Island) and cover a large geographic area. Their exact locations were required to be anonymous as per agreements with individual plants. Details of these plants were described in previous studies (Kim et al. 2013a, 2013b). They employ various methods, mainly anaerobic digestion, to convert raw sludge to biosolids. The populations served and the wastewater flow rates were provided by plant personnel (Table 1). In addition to domestic wastewater, the eight plants also receive wastewaters from industrial, commercial, and institutional facilities and are representative of secondary plants in Canada.

**Table 1:** Features of WWTPs selected

Code	Biological treatment	Biosolids production method	Wastewater flow (ML/d)	Population served (10^3^ persons)
1	BAF	Mesophilic anaerobic digestion	61	105
2	AS	Mesophilic anaerobic digestion	22	32
3	AS	Mesophilic anaerobic digestion	410	1000
4	AS	Mesophilic anaerobic digestion	340	480
5	AS	Mesophilic anaerobic digestion	190	374
6	AS	Aerobic digestion	2.6	3.0
7	AS	Alkaline treatment	21	45
8	TF	Thermophilic anaerobic digestion	560	900

### Chemical substances

Five BFRs (Table 2) were measured at the eight plants. The first three (BDE-209, 99 and 47) are legacy PBDEs used in plastics, textiles, and many other products (Darnerud 2003). The other two are PBDE replacements with BTBPE being used in plastic housing for electronics (Renner 2004) and PBEB in polyurethane foam and thermoset polyester resins for circuit boards, textiles, adhesives, wire, and cable coatings (Covaci et al. 2011). They were selected in this study because their environmental risks have not been examined in Canada and wastewater related data are an important part of data requirements for risk assessment. There are other PBDE replacements on the market such as tretrabromobisphenol A (TBBPA), bis(2-ethylhexyl)-2,3,4,5-tetrabromophthalate (TBPH), and 2-ethylhexyl-2,3,4,5 tetrabromobenzoate (TBB). They have been evaluated by Environment Canada and Health Canada (2013, 2016) and are, therefore, not prioritized for this study.

**Table 2:** Flame retardants measured at WWTPs

Acronym	Name	CASRN	Formula
BDE-209	2,2′,3,3′,4,4′,5,5′,6,6′-decabromodiphenyl ether	1163-19-5	C_12_Br_10_O
BDE-99	2,2′,4,4′,5-pentabromodiphenyl ether	60348-60-9	C_12_H_5_Br_5_O
BDE-47	2,2′,4,4′-tetrabromodiphenyl ether	5436-43-1	C_12_H_6_Br_4_O
BTBPE	1,2-bis(2,4,6-tribromophenoxy) ethane	37853-59-1	C_14_H_8_Br_6_O_2_
PBEB	Pentabromoethylbenzene	85-22-3	C_8_H_5_Br_5_

### Sampling and measurement

Sampling was conducted from 2009 to 2011 (see sampling dates in Table S1 in supplementary data). It was divided into two seasons: warm (July, August, September, and October) and cold (January, February, March, and December). No sampling was conducted in April, May, June, and November. For plants 1 and 2, four sampling trips were made over 2 years (two warm seasons and two cold seasons) and samples were collected for three consecutive days per plant on each trip (a total of 12 sampling days per plant). For each of the remaining 6 plants, two sampling trips were made within a 1-year period (one warm season and one cold season) with three consecutive days of sampling on each trip (a total of 6 sampling days per plant).

The streams sampled included influent, final effluent, and biosolids (Fig. 1). For plants 1, 3, 4, and 8, final effluent samples were taken prior to chlorine disinfection in order to reduce potential chemical interferences in sample analysis (Kim et al. 2013a). The other four plants (2, 5, 6, and 7) employed ultraviolet light for disinfection and the final effluent was sampled after disinfection.

Bulk liquid (influent and final effluent) samples were collected as 24-h equal volume composites (400 mL every 30 min) on each sampling day. Grab samples were collected for biosolids on each sampling day. Grab samples are considered representative of the biosolids stream sampled because clarifier and digester retention times reduce variability and produce a more homogeneous mixture. These sampling practices are commonly used for wastewater liquid and solids streams (Katsoyiannis and Samara 2005). Samples were stored at 4 °C, transported to Axys Analytical Services (Sidney, BC) on ice by overnight courier and analyzed within 2 weeks.

The concentrations of the five flame retardants in liquid and biosolids samples were measured according to USEPA (2007) Method 1614A, described elsewhere (Kim et al. (2013a, b and 2014). Briefly, liquid and biosolids samples (1 L and 5 g dry weight, respectively) were spiked with isotopically labeled surrogate standards (^13^C_12_-BDE-47, ^13^C_12_-BDE-99, and ^13^C_12_-BDE-209 in PBDE measurements and ^13^C_12_-BTBPE in BTBPE measurements; no surrogate available at the time of the analysis for PBEB). The spiked liquid samples were extracted by liquid-liquid extraction and the spiked biosolids samples by Soxhlet extraction, both with dichloromethane. The extracts were spiked with a cleanup standard (^13^C_12_-BDE-139) and cleaned up by fractionation using three different columns: multi-layered acid/base silica, florisil, and alumina. The extracts were then concentrated to 20 μL with the addition of injection standards (^13^C_12_-BDE-79, ^13^C_12_-BDE-180, and ^13^C_12_-BDE-206). Finally, the extracts were analyzed using high-resolution gas chromatography (Hewlett Packard 6890 with a DB-5HT capillary column of 30-m long, 0.25-mm inside diameter, and 0.1-μm film thickness)/high resolution mass spectrometry (Micromass Ultima tuned to a static mass resolution of 5000 and calibrated using PFK as reference). The quality assurance and quality control used during the measurements included field equipment blanks, laboratory blanks, laboratory duplicates, and spiked blanks. All standard solutions used for analysis such as labeled surrogate, labeled cleanup, labeled recovery, authentic spike, and calibration solution were prepared by diluting aliquots of stock solutions with toluene. Recovery efficiencies for labeled surrogate standards were 74–90%. At least one procedural blank was analyzed within each batch. Measured blank concentrations were one or two orders of magnitude lower than measured concentrations in samples and were not subtracted in calculating concentrations in influent, effluent, and biosolids.

The total suspended solids (TSS) in liquid samples were determined at Environment and Climate Change Canada’s (ECCC) National Laboratory for Environmental Testing according to standard methods (APHA, AWWA, and WPCF 2005). Liquid samples were filtered using pre-weighed Whatman 934-AH glass microfiber filters (Thermo Fisher Scientific, Waltham MA, USA). The filter cake including the filter was dried in an oven at 105 °C for over 24 h and re-weighed. The weight of the dried cake was determined by subtracting the filter weight and divided by the volume of a liquid sample to calculate the TSS concentration.

The reporting limits (RLs) of the chemical analyses are sample specific (Table 3). These limits account for the influence of matrix of each sample. They are calculated as three times the chromatographic noise of each analysis run. The wide range of these limits indicates the complexity of matrix in wastewater samples.

**Table 3:** Reporting limits for flame retardants

Flame retardant	RL for liquid samples (ng/L)	RL for biosolids samples (ng/g)
BDE-209	0.011–2.6	0.089–6.4
BDE-99	0.0007–0.058	0.048–0.64
BDE-47	0.0002–0.010	0.001–0.024
BTBPE	0.002–1.1	0.099–13
PBEB	0.0003–0.012	0.0009–0.25

## Results and discussion

### Flame retardant concentrations and removal rates

Concentrations of flame retardants in this study are total concentrations, i.e., samples were analyzed without separation of the particulate phase from the aqueous phase. However, concentrations in final effluent were mainly associated with the aqueous phase due to the low content of particles. Concentrations in biosolids were, on the other hand, mainly associated with the particulate phase due to the combination of the high particulate content and the hydrophobic nature of the five flame retardants. The detection frequency of all influent, effluent, and biosolids samples from the eight plants combined was 100% for the three PBDE congeners, 97% for BTBPE, and 91% for PBEB. Concentrations below RLs (non-detects) were estimated as one half of RLs. The overall rate of non-detects was less than 3% of all measurements.

Table 4 summarizes the ranges of the measured concentrations of the five flame retardants from the eight plants. The ranges at a plant spanned approximately one order of magnitude for each stream (influent, final effluent, and biosolids). The concentration differences between the eight plants were large in the range of one to two orders of magnitude. At least one-order-of-magnitude variations were also reported for influent PBDEs and other organic compounds within the same WWTP or between different WWTPs in Norway (Vogelsang et al. 2006). The influent concentrations of the three legacy PBDEs (BDE-209, 99, and 47) were on the order of 10 to 100 or several 100s of ng/L from the eight plants and lower for the two replacements (BTBPE and PBEB). The final effluent concentrations of the five flame retardants showed significant reductions from the influent and indicate substantial removal during treatment. Previous studies in Canada (Kim et al. 2013a, 2013b and Kim et al. 2014) covered a wide range of WWTPs including not only secondary plants, but also lagoons and chemically assisted primary plants. These studies found that BDE-209, 99, and 47 were most prevalent, accounting for more than 70% of 30 plus frequently detected PBDE congeners in influent, effluent, and biosolids. The concentrations of those congeners, BTBPE and PBEB, in influent (21–1000, 0.7–16, and 0.007–0.1 ng/L, respectively) and biosolids (420–6000, 8–420, and 0.05–0.29 ng/g, respectively) were in line with those given in Table 4. This implies that the eight secondary plants selected in this study were reflective of various types of wastewater treatment in Canada with respect to the five flame retardants in influent and biosolids (not applicable to lagoons). However, those studies showed higher upper-end effluent concentrations (3–270, 0.12–1.7, and 0.001–0.26 ng/L for PBDEs, BTBPE, and PBEB, respectively) compared to those from the eight secondary plants in Table 4. This suggests higher effluent releases of the five flame retardants from lagoons and/or chemically assisted primary plants. The three PBDE congeners were also prevalent among all PBDEs detected at WWTPs in the US (Venkatesan and Halden 2014), Australia (Law and Herzke 2011), and Korea (Lee et al. 2014). The concentrations of BDE-209, 99, and 47 in the final effluents of the eight secondary plants were in line with the averages (1.7, 11.2, and 10.5 ng/L, respectively) found at a secondary plant in California, US (North 2004). The concentrations of BDE-209 in biosolids were on the same order of magnitude (hundreds to thousands ng/g) as reported from 17 WWTPs in Spain (Gorga et al. 2013), but below the average (6870 ng/g) measured in 2006–2007 from plants in Chicago, US (Hale et al. 2012). The concentration ranges for BTBPE and PBEB in biosolids were significantly lower than those (37–9060 ng/g and 0.77–2.5 ng/g) found in biosolids samples collected in 2001 from 94 US WWTPs (Venkatesan and Halden 2014).

**Table 4:** Flame retardant concentrations from eight WWTPs (median in parentheses)

Flame retardant	Influent (ng/L)	Final effluent (ng/L)	Biosolids (ng/g)
BDE-209	16–805 (64)	1.3–36 (3.1)	276–3540 (1055)
BDE-99	11–78 (33)	0.3–9.7 (3.3)	173–645 (357)
BDE-47	11–74 (33)	0.8–11 (3.7)	183–667 (359)
BTBPE	0.07–11 (1.2)	0.005–0.8 (0.11)	6.6–316 (13.5)
PBEB	0.002–0.06 (0.011)	0.0002–0.005 (0.0009)	0.03–0.26 (0.086)

**Fig. 2:**
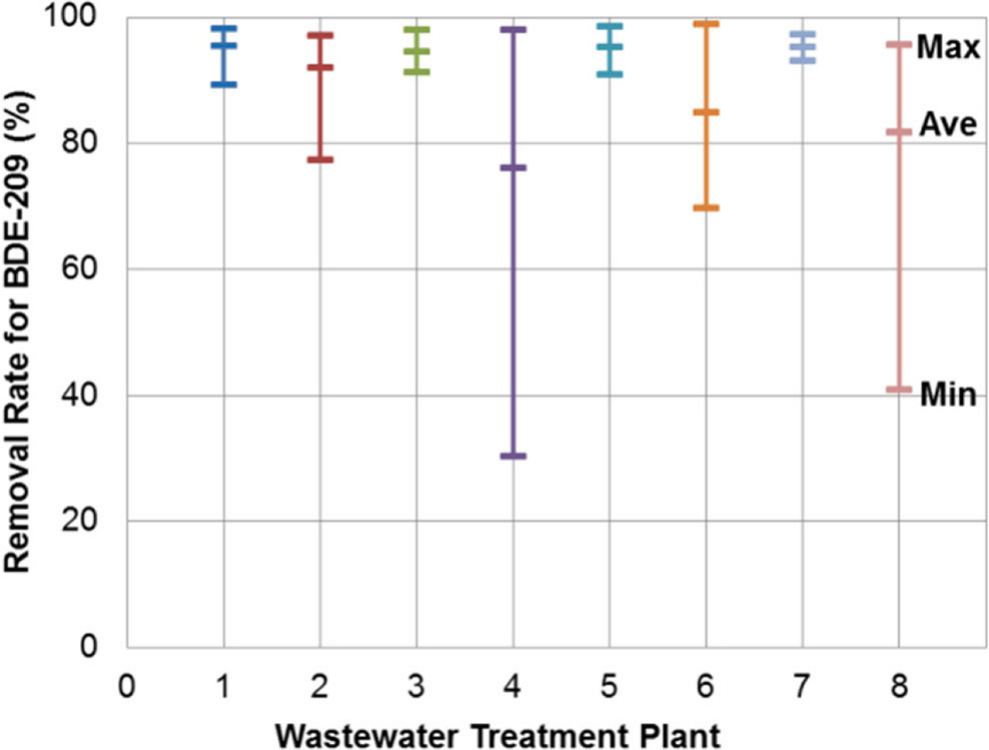
Removal rate for BDE-209 at eight wastewater treatment plants (The range of the removal rate is based on 12 data points for plants 1 and 2, and 6 data points for plants 3–8)

The removal rate of each flame retardant was calculated from influent and final effluent concentrations. Figure 2 illustrates the ranges and averages of the removal rate for BDE-209 at the eight plants and Figs. S1–4 (supplementary data) for the other four flame retardants. These ranges reflect the degree of variability within a plant and are grouped into three categories: low (absolute magnitude of variation < 10%), moderate (10–20%), and high (> 20%). For plant 1 (BAF), the removal rate variability was moderate for BDE-209, BDE-99, BDE-47, and BTBPE, but high (nearly 30%) for PBEB though the same samples were used in the analysis of the five flame retardants. For each of the six AS plants (2–7), the variability was not found to follow a consistent pattern between the five flame retardants and appeared to be of a random nature. For example, a low degree of variability was observed for BDE-209 at plant 3, but a high one (40 to 80%) for the other four. For plant 8 (TF), the five flame retardants all showed high variability (30 to 50%). It appeared that the degree of the removal rate variability was not uniform between the five flame retardants within a plant. This might be due to the variations of their influent concentrations or loadings considering each flame retardant had its own origin and release pattern. The plant-to-plant variability can be indicated by the differences in the average removal rate between the eight plants. These differences are found not to be significantly different from the variability within a plant. They are further found not to be associated with specific treatment processes (BAF for plant 1, AS for plants 2–7, and TF for plant 8). This process-independent feature might be related to the recalcitrant nature of a substance which is insensitive to biological treatment. The same was also reported in a study of several US plants that showed no apparent influence of different biological processes on the removal of PBDEs (Rocha-Gutierrez and Lee 2013). Although recalcitrant substances are insensitive to biological treatment, their removal rates can still be affected by biological treatment conditions (e.g., biomass concentration in bioreactors) through mechanisms other than biodegradation (e.g., sludge sorption). Thus, the removal variability of recalcitrant substances is expected to reflect the variability in biological treatment conditions though not as much as for biodegradable substances.

The removal of the five flame retardants is mainly due to their sorption to sludge. The degree of the sorption can be indicated by the octanol-water partition coefficients (*K*_ow_) of the five flame retardants, a measure for hydrophobicity. Their log *K*_ow_ values are predicted to be in the range of 6.8–12.1 (BDE-209, 12.1; BDE-99, 6.8; BDE-47, 6.8; BTBPE, 9.2; PBEB, 7.5 from EPI Suite 2012). These values indicate high hydrophobicity or sorption to sludge. They are in line with the log *K*_ow_ range of 6–10 reported for PBDEs (deca-BDE, 10; octa-BDE, 8.4–8.9; penta-BDE, 6.6–7.0; tetra-BDE, 5.9–6.2 from Guerra et al. 2011; BDE-99, 7.3; BDE-47, 6.8 from Braekevelt et al. 2003) and the two novel BFRs (BTBPE, 7.9 and PBEB, 6.4 from Covaci et al. 2011). In a WWTP, biodegradation and volatilization are two common removal mechanisms besides sludge sorption. According to model predictions (EPI Suite 2012), the five flame retardants are recalcitrant and low in volatility, with low Henry’s Law constants in the range of 0.005 to 11 Pa-m^3^/mol (BDE-209, 0.005; BDE-99, 0.36; BDE-47, 0.86; BTBPE, 0.043; PBEB, 11). The low volatility was demonstrated by measurements that showed a very small fraction (0.01%) of influent BDE-209, 99, and 47 released to air (Martellini et al. 2012). These properties rendered sludge sorption to be the major route for the removal of the five flame retardants.

**Fig. 3:**
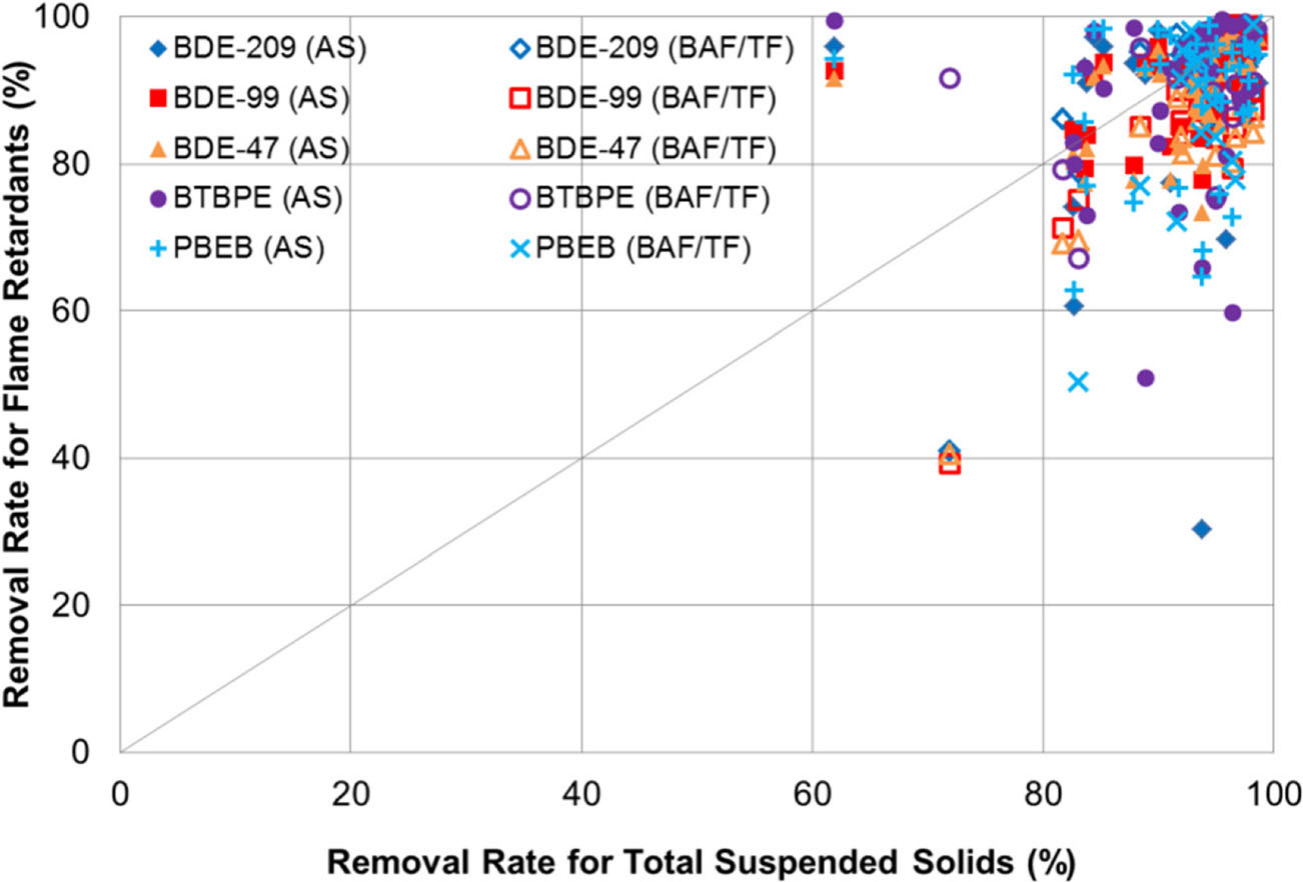
Removal of five flame retardants vs. removal of total suspended solids (Each removal rate for flame retardants is determined from a pair of 24-h composite influent and effluent samples. The corresponding TSS removal rate is based on the same samples. AS, activated sludge; BAF/TF, biological active filter or trickling filter)

The removal of the five flame retardants is therefore expected to correspond to the removal of TSS, as indicated in Fig. 3. The TSS concentrations measured from the eight plants were in the range of 41–266 mg/L in influent and reduced to 3–70 mg/L in final effluent (Table 5). The TSS removal rate was high: predominantly between 80 and 99%. The removal rate for each flame retardant was also high with the majority of data points in the range of 70–99%. Because of their recalcitrant, hydrophobic, and low-volatility characteristics, the fate of the five flame retardants was governed by the fate of TSS. Figure 3 shows the correspondence between the high removal of the five flame retardants and the high TSS removal. The figure is not intended to correlate the two parameters because the TSS removal range is not wide enough and does not contain low or medium data points. Song et al. (2006) showed the role of TSS in PBDE removal from their measurements at a Canadian WWTP. They reported that the transfer to sludge of several major PBDE congeners including BDE-99 and 47 was 53% via primary sedimentation at 73% TSS removal and increased to 91% when 95% of the TSS in the final effluent was removed. In Fig. 3, the data points from the AS process (plants 2–7) are presented separately, but show no segregation from the BAF (plant 1) and TF (plant 8) processes. This demonstrates the insensitivity of the removal of the flame retardants to different types of biological treatment due to their recalcitrant nature.

**Table 5:** Total suspended solids (TSS) in influent and effluent (median in parentheses)

Plant	Influent TSS (mg/L)	Effluent TSS (mg/L)
1	113–174 (147)	3–17 (9)
2	72–202 (135)	3–12 (4)
3	54–170 (98)	6–70 (10)
4	113–266 (127)	12–23 (19)
5	128–222 (167)	6–24 (11)
6	41–240 (105)	6–7 (7)
7	74–236 (144)	6–14 (9)
8	64–182 (145)	6–21 (14)

### Release-characterizing parameters

Although a substance’s removal rate is often used in calculating its releases to receiving water, the release fraction is a more direct parameter for the calculation. It also provides better precision and easier interpretation than the removal rate when the latter approaches 100%. In a similar manner, the amount of a substance transferred from influent to biosolids, here referred to as influent-biosolids transfer coefficient, is a key parameter in determining releases to soil via biosolids land application. The two parameters are defined as:

1 $$ {E}_{\mathrm{f}}=\frac{C_{\mathrm{f}}}{C_{\mathrm{i}}} $$

2 $$ {K}_{\mathrm{i}\mathrm{b}}=\frac{C_{\mathrm{b}}}{C_{\mathrm{i}}} $$

where

*E*_f_: Release fraction of a substance to final effluent, unitless
*K*_ib_: Influent-biosolids transfer coefficient of a substance, L/g
*C*_i_: Concentration in influent, ng/L
*C*_f_: Concentration in final effluent, ng/L
*C*_b_: Concentration in biosolids, ng/g

For each flame retardant-WWTP combination, its release fractions and influent-biosolids transfer coefficients were calculated based on the samples collected on the same day. In general, concentrations measured in influent, final effluent, and biosolids do not necessarily match with one another hydraulically due to hydraulic retention times (HRTs). This can result in inaccurate determination of the two parameters in the event of variation. However, 24-h composite samples accommodate variation. Grab samples are common for biosolids which are, by their nature, composite entities as discussed above. Long retention times for biosolids (normally weeks) are expected to yield relatively small day-to-day variations of the concentrations of the five flame retardants in biosolids. The variations of the influent-biosolids transfer coefficient so calculated would, therefore, reflect mainly the variations of the influent concentrations and may not be used as an indicator for the day-to-day variations of the concentrations in biosolids. However, this limitation is minimal for the seasonal variations of the coefficient where the sampling timeframes were much longer than biosolids retention times.

### Intra-plant release variability

Two plants (1 and 2) were selected for intra-plant variability analysis (within a plant) because samples were collected over a longer period (18–24 months) compared to the other six plants (7–10 months). These long-term multi-season samples allow for an analysis of release variability with time, in particular under different seasonal conditions, at a plant. The other six plants also showed variability with time, but the data from the first two plants is more extensive and is better suited for intra-plant variability analysis.

**Fig. 4:**
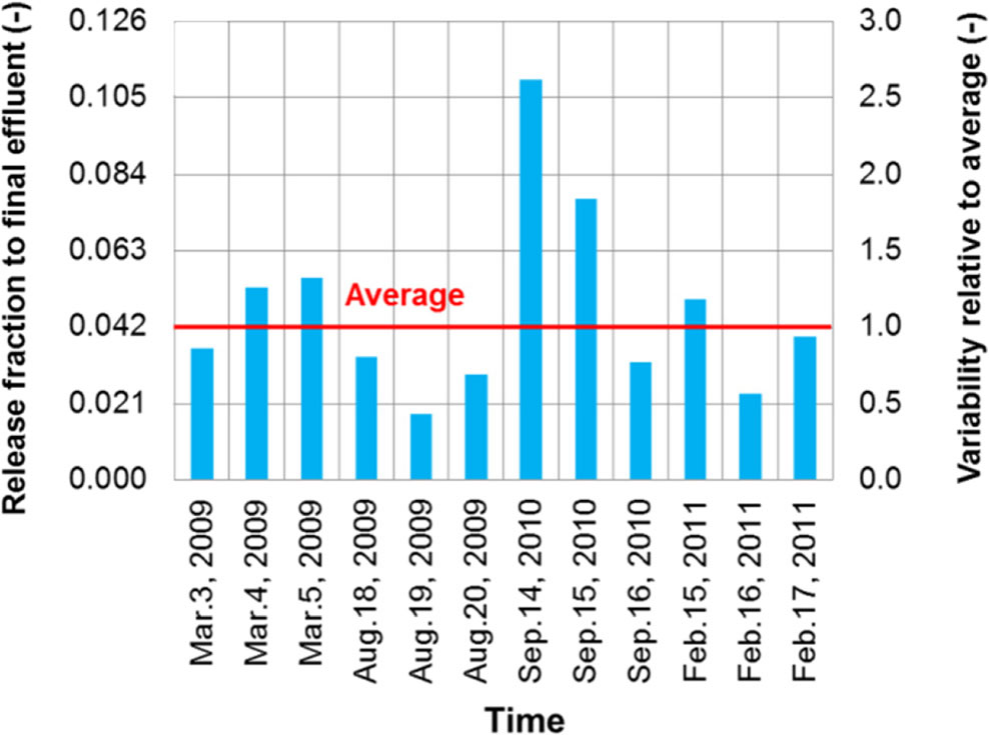
Intra-plant variability for release fraction of BDE-209 to final effluent at plant 1 (The variability of the release fraction is quantified by comparing its individual values to a temporal average)

The intra-plant variability was analyzed with respect to the two release-characterizing parameters (release fraction and influent-biosolids transfer coefficient). Individual values were compared with their averages as a way to characterize the variability. For example, 12 values corresponding to 12 sampling days over a 24-month period were obtained for the release fraction of BDE-209 to the final effluent of plant 1 (Fig. 4). These values were in the range of 0.018 to 0.109 with an average of 0.042; variability was in the range of 0.4–2.6, calculated as the ratio of each value to the average.

**Fig. 5:**
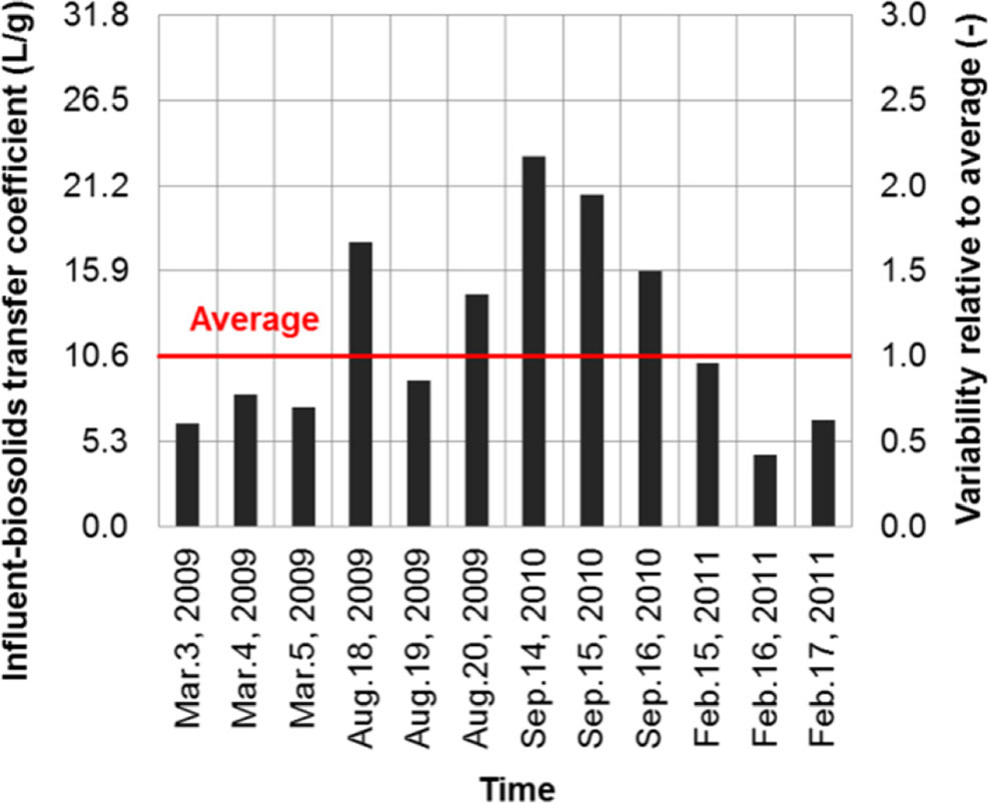
Intra-plant variability for influent-biosolids transfer coefficient of BDE-209 at plant 1 (The variability of the influent-biosolids transfer coefficient is quantified by comparing its individual values to a temporal average)

Another example is the variability of the influent-biosolids transfer coefficient for BDE-209 also at plant 1 (Fig. 5). Twelve values in the range of 4–23 L/g were obtained for the coefficient. Their average was 10.6 L/g. The variability was calculated in the range of 0.4–2.2 as the ratio of each value to the average.

**Fig. 6:**
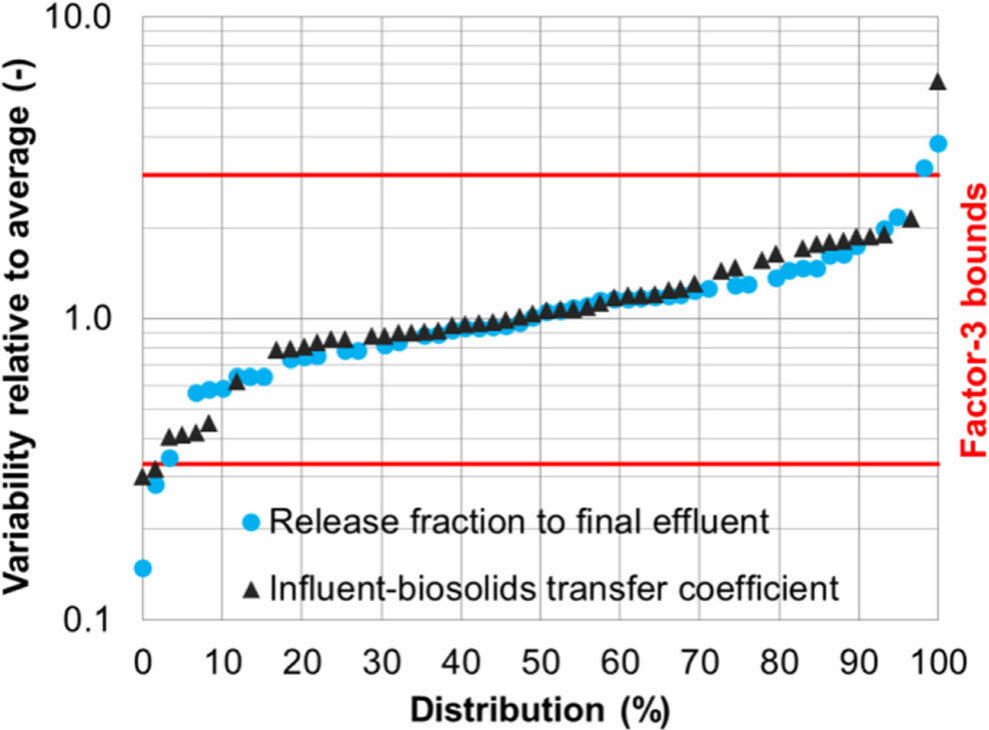
Intra-plant variability relative to averages for all flame retardants at plant 1 (Each data point associates with one flame retardant on a given sampling day)

The intra-plant variability of the two release-characterizing parameters was found to range from 0.3 to 3 for plant 1 when all five flame retardants were considered (Fig. 6). In other words, the release fraction of a flame retardant to final effluent, or the influent-biosolids transfer coefficient fell within a range bounded by a factor of 3 above and below its average. This factor-3 variability is observed for both plants 1 and 2 for the majority (> 90%) of the data points. Intra-plant variability was also reported for a non-biodegradable substance (octylphenol) and its release fraction to final effluent at a WWTP varied between 0.01 and 0.14 within a 2.5 year period (Hohne and Puttmann 2008). This temporal variation was over one order of magnitude which is in agreement with the observed factor-3 variability in this study.

The factor-3 variability of the release fraction was a result of the concentration variations of the five flame retardants in both influent and effluent. These variations occurred on short- and long-term basis. The short-term variations could be as large as fivefold from 1 day to another within a given three consecutive day sampling period at plants 1 and 2. The long-term variations could be larger up to tenfold when 24-h composite influent or effluent concentrations from one season were compared to those from another season. The observed factor-3 variability was the combined outcome of these short- and long-term variations.

The factor-3 variability of the influent-biosolids transfer coefficient was also a result of concentration variations. The day-to-day variations of the five flame retardants in biosolids were not significant (within 20%) within a given three consecutive day sampling period. The variability of the influent-biosolids transfer coefficient during such a period resulted mainly from influent concentration variations. The season-to-season concentration variations of the five flame retardants in biosolids were up to tenfold and comparable to those in influent. These long-term variations combined with short-term influent concentration variations contributed to the variability of the influent-biosolids transfer coefficient.

The observed factor-3 variability has an important bearing on a substance’s concentration in final effluent. For the five flame retardants measured at plants 1 and 2, the average release fraction to final effluent was in the range of 0.03–0.12, so the release fraction on a given day could vary from a low of 0.01 to a high of 0.36 in light of the factor-3 variability. However, if the average release fraction of a substance goes beyond 0.33 and the factor-3 variability is presumably still valid, the release fraction of the substance can be larger than one during certain time periods with the final effluent concentration exceeding the influent. Although mathematically possible, this hypothetic case is highly unrealistic. The degree of the variability is therefore expected to decrease at a higher release fraction. The domain of applicability for the observed factor-3 variability should be limited to the specific physical-chemical properties of the five flame retardants.

### Inter-plant release variability

The inter-plant variability (between different plants) of the two release-characterizing parameters was quantified with respect to overall averages. An overall average release fraction or influent-biosolids transfer coefficient is the average of all averages across the eight plants for a given flame retardant. As summarized in Table 6, the averages of the two parameters for each flame retardant varied by up to one order of magnitude from one plant to another. Variability up to one order of magnitude has also been reported for the release fractions of many fatty and resin acids from different activated sludge WWTPs used at pulp and paper mills (LaFleur et al. 1997). The overall averages of the two parameters showed differences within a factor of 2 between the five flame retardants, 0.08–0.13 for release fraction and 7–14 L/g for influent-biosolids transfer coefficient. These differences could be a result of the different physical-chemical properties of the five flame retardants, primarily hydrophobicity which is the major driver for the fate of non-volatile, non-biodegradable substances in wastewater treatment.

**Table 6:** Release-characterizing parameters for eight wastewater treatment plants

Flame retardant	Overall average (range of plant-specific averages)	
	Release fraction to final effluent (−)	Influent-biosolids transfer coefficient (L/g)
BDE-209	0.078 (0.036–0.134)	13.8 (2.6–23.7)
BDE-99	0.103 (0.015–0.216)	11.2 (5.0–17.4)
BDE-47	0.119 (0.034–0.229)	11.2 (4.9–16.1)
BTBPE	0.096 (0.029–0.154)	13.9 (8.9–25.9)
PBEB	0.132 (0.063–0.285)	7.3 (4.0–10.7)

**Fig. 7:**
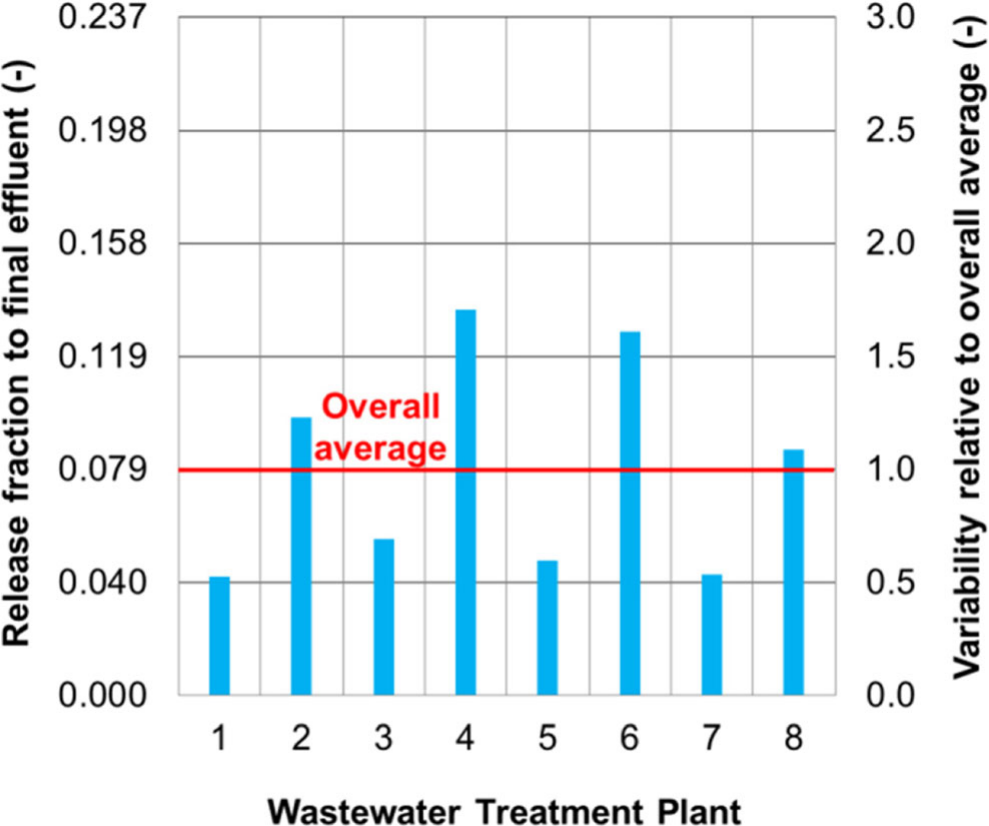
Inter-plant variability for BDE-209 in final effluent (The release fraction at a plant is a temporal average based on all samples collected from the plant and the overall average is the spatial average across all eight plants)

The inter-plant variability was quantified by comparing the average release fraction or influent-biosolids transfer coefficient of a flame retardant at a plant to its overall average across the eight plants. Figure 7 illustrates this quantification for the release fraction of BDE-209 to final effluent. Its averages from the eight plants were in the range of 0.042–0.13 with an overall average of 0.079. The inter-plant variability was then determined to be in the range of 0.5–1.7 relative to the overall average. The inter-plant variability of the influent-biosolids transfer coefficient was determined in the same manner.

**Fig. 8:**
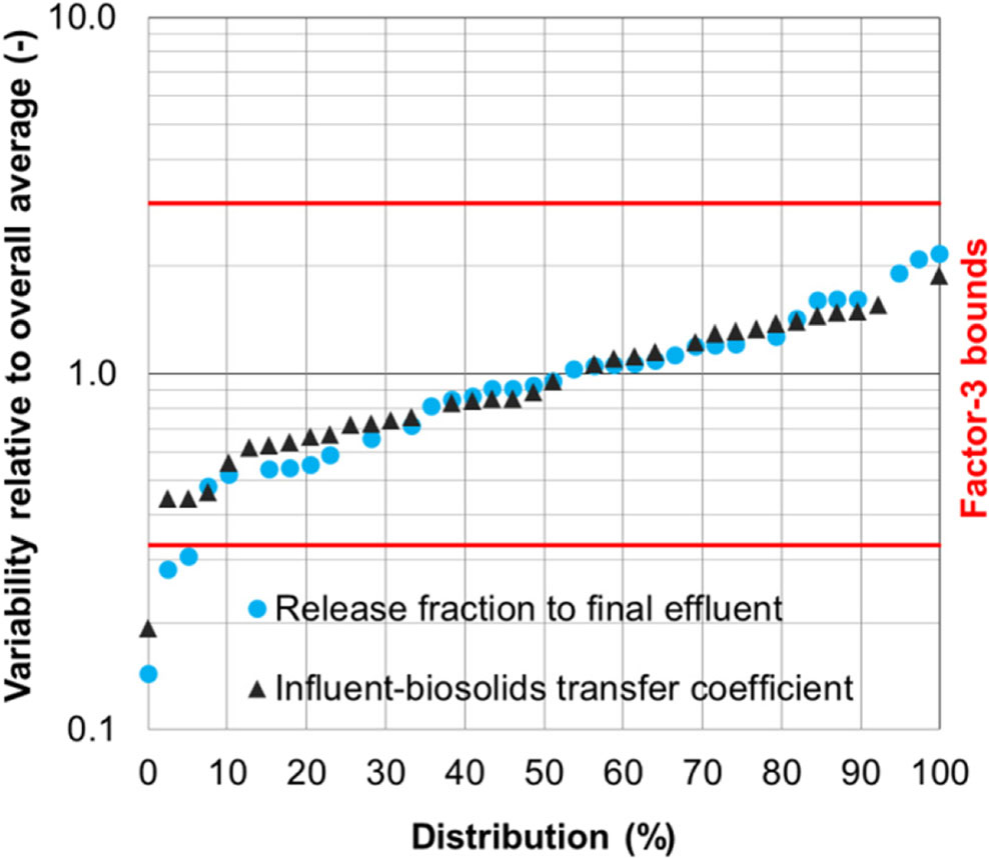
Inter-plant variability relative to overall averages for all flame retardants (Each data point associates with one flame retardant averaged over samples collected at a given plant)

The range of the inter-plant variability was within 0.3–3 for the two release-characterizing parameters when all five flame retardants were considered (Fig. 8). The range covered the majority of the data points (> 90%) and was the same as the intra-plant variability observed for plants 1 and 2. This indicates that the differences between different plants were comparable to the day-to-day or season-to-season variations of a single plant with respect to release fraction and influent-biosolids transfer coefficient. These plants were not limited to one process (activated sludge) and included biological active filter and trickling filter. Jose et al. (Joss et al. 2005) reported the same phenomenon for the release fractions of several pharmaceuticals and fragrances in final effluent. They found that the differences in these release fractions between three processes studied (activated sludge, membrane bioreactor, and fixed-bed bioreactor) were comparable to those from the activated sludge process and could not be explained by the differences of the three processes. As discussed previously in Fig. 3, the three biological treatment processes used by the eight plants in this study showed little difference in the removal of the five flame retardants. The inter-plant variability results of the two release-characterizing parameters further indicated that the three processes yielded the same or similar release fractions to effluent and the same or similar concentrations in biosolids per unit influent concentration. From these results, it may be inferred that each process did not have particular advantage over the other two with regard to the treatment of the five flame retardants. However, this inference only pertains to the five flame retardants studied, although it may be extended to other substances of the same nature.

The plant-to-plant variability observed in this study could be attributed to different influent flame retardant and TSS concentrations and plant operating conditions. The influent flame retardant concentrations differed by one to two orders of magnitude between the eight plants. The median influent TSS (Table 5) varied from 98 mg/L (plant 3) to 167 mg/L (plant 5). The differences in operating conditions between the plants were less than one order of magnitude: 4–9 days for sludge age or sludge retention time (SRT), 1–5 g/L for mixed liquor suspended solids, 1–7 h for primary clarifier hydraulic retention time (HRT), 2–15 h for bioreactor HRT, and 3–10 h for secondary clarifier HRT (Kim et al. 2013a). These different flame retardant and TSS concentrations and operating conditions are believed to contribute to different extents of releases to final effluent and transfers to biosolids. However, a quantitative analysis of the contribution cannot be warranted because the size of the dataset from this study is not sufficiently large. A study by Douziech et al. (2018) presented a regression analysis of removal rate based on a large dataset of over 200 chemicals measured in activated sludge plants from 28 countries. The study determined sludge age to be important and showed statistically an upward trend for removal rate with SRT increasing from 2 to 50 days. The SRTs of the eight plants were within a much narrow range (4–9 days) which is typical of Canadian treatment conditions and might not be wide enough to allow for the trend analysis of release fraction or influent-biosolids transfer coefficient.

The eight plants selected are considered to be adequate for inter-plant variability analysis. The averages for release fraction and influent-biosolids transfer coefficient were in the ranges of 0.02–0.29 and 3–24 L/g, respectively, for the five flame retardants from the six plants (3 through 8) with 7–10 month sampling periods. These ranges remained the same when the other two plants (1 and 2) with longer sampling periods (18–24 months) were added except for one slightly higher value of 26 L/g for influent-biosolids transfer coefficient. This suggests that the observed factor-3 inter-plant variability is likely to remain unchanged if more plants are added and is thus expected to reflect the spatial variability across a large number of secondary plants in Canada. However, the observed factor-3 variability should be considered to be applicable to the five flame retardants or substances with similar properties.

### Implications for ecological exposure assessment

The purpose of ecological exposure assessment is to determine the level of exposure to a chemical substance in an environmental compartment so the risk posed by the substance to ecological organisms can be evaluated. WWTPs are a common pathway for many substances to enter receiving water via effluent discharge and soil via biosolids land application. The levels of the resulting aquatic and soil exposure are in direct proportion to a substance’s concentrations in final effluent and biosolids, respectively (ECHA 2016). These concentrations are functions of the two release-characterizing parameters (release fraction and influent-biosolids transfer coefficient) analyzed in this study.

The values of the two parameters obtained for the five flame retardants are expected to be a good approximation for substances with similar properties. This is because the fate of a substance in wastewater treatment is governed by its physical-chemical properties, mainly hydrophobicity, biodegradability, and volatility. The five flame retardants are hydrophobic, recalcitrant, and low in volatility according to model predictions (EPI Suite 2012). Substances with these characteristics can be expected to have a similar fate in wastewater treatment with averages for release fraction and influent-biosolids transfer coefficient similar to those for the five flame retardants, 0.02–0.29 and 3–26 L/g, respectively. The spread of these values is about one order of magnitude and could be attributed to different plant operating conditions as well as variations in a substance’s influent concentrations and physical-chemical properties. The effect of those properties such as hydrophobicity, molecular weight, and water solubility on the two parameters was not investigated due to the limited number (5) of substances studied.

The observed degree of intra- or inter-plant variability can assist in exposure variability analysis. When release fraction and influent-biosolids transfer coefficient are the primary factors causing exposure variability, the possible ranges of exposure are expected to be within a factor-3 envelope for hydrophobic, non-biodegradable, and non-volatile substances. More specifically, the level of exposure in receiving water or soil resulting from a single plant can vary temporally to an extent of up to three times below or above its average. The same degree of variability is also expected for a spatial exposure profile across WWTPs in different geographic locations. In reality, a higher degree of variability in exposure may occur if other contributing parameters such as influent loading vary substantially.

The observed factor-3 variability can further help interpret the results of exposure characterization for substances undergoing detailed evaluation. Although the exposure results are often expressed as fixed numerical values, they should be interpretated to be able to vary to a certain range in light of variability. The observed factor-3 variability provides empirical evidence for this interpretation. For many substances, measured data for release fraction and influent-biosolids transfer coefficient can be limited and model estimates can be very approximative due to insufficient physical-chemical properties. These circumstances increase the uncertainty in exposure characterization. The exposure results are expected to diverge more when the uncertainty adds on the top of the observed factor-3 variability.

The observed factor-3 variability also offers much needed data for screening-level exposure analysis when prioritizing substances for risk assessment or determining if a substance deserves a more detailed evaluation. In the analysis, parameters key to exposure are assigned conservative values under a worst case scenario. These parameters include release fraction to final effluent, influent loading and receiving water dilution for aquatic exposure, and influent-biosolids transfer coefficient, influent loading, biosolids application rate, years of accumulation, and soil till depth for soil exposure (ECHA 2016). Conservative values can be readily determined for these parameters except for release fraction and influent-biosolids transfer coefficient. The difficulty with the two parameters is due to their dependence on varying plant operating conditions. To resolve this difficulty, averages can be first estimated for the two parameters using models under average plant conditions and their conservative values can be readily determined by applying the factor-3 variability found from this study.

## Conclusions

Release fraction to final effluent and influent-biosolids transfer coefficient are two release-characterizing parameters important to ecological exposure assessment for aquatic and soil compartments, respectively. Averages for release fraction were determined to be in the range of 0.02–0.29 for five flame retardants (BDE-209, BDE-99, BDE-47, BTBPE, and PBEB) from eight secondary WWTPs and those for influent-biosolids transfer coefficient in the range of 3–26 L/g. These ranges are expected to be applicable to and, therefore, provide read-across data for the exposure assessment of similar substances which are recalcitrant, low in volatility, and high in hydrophobicity.

The two parameters for the five flame retardants were found to vary within a factor of 3 above or below their averages with time at a WWTP and spatially across different WWTPs. The observed factor-3 variability offers an empirical rule of thumb for use in characterizing the temporal and spatial variations of the environmental releases of the five flame retardants and other similar substances. The factor-3 variability can also be used in screening-level exposure analysis under worst case scenarios. In this, conservative values for release fraction and influent-biosolids transfer coefficient can be readily determined using a rule of 3 times model-estimated averages.

## Electronic supplementary material

ESM 1 (DOCX 100 kb)
