# Supplementary material for: Variability of release rate of flame retardants in wastewater treatment plants
Source: Environ Sci Pollut Res Int. 2018 Oct 15;25(34):34740–52. doi: 10.1007/s11356-018-3403-2 (PMC6245005; doi:10.1007/s11356-018-3403-2)
Supplement: Supplementary file 1 — (DOCX 100 kb) [file 11356_2018_3403_MOESM1_ESM.docx]

Supplementary Data

Variability of Release Rate of Flame Retardants in Wastewater Treatment Plants

*Jesse Shen^*,†^, Shirley Anne Smyth^†^, Ronald Droste^‡^ and Danaëlle Delâge^†^*

^†^Science and Risk Assessment Directorate, Science and Technology Branch, Environment and Climate Change Canada, 351 Saint Joseph Boulevard, Gatineau, Quebec, Canada K1A 0H3

^‡^Department of Civil Engineering, University of Ottawa, Ottawa, Ontario, Canada K1N 6N5

^*^Corresponding author, email: [jesse.shen@canada.ca](mailto:jesse.shen@canada.ca)

**Table S1 – Sampling Dates of Eight Wastewater Treatment Plants**

| Season | Date | Plant |
| --- | --- | --- |
| Cold | February 10-12, 2009 | 3 |
| Cold | February 17-19, 2009 | 6 |
| Cold | March 3-5, 2009 | 1 |
| Cold | March 10-12, 2009 | 5 |
| Warm | July 7-9, 2009 | 2 |
| Warm | July 14-16, 2009 | 7 |
| Warm | July 21-23, 2009 | 8 |
| Warm | August 18-20, 2009 | 1 |
| Warm | August 25-27, 2009 | 5 |
| Warm | September 29-30 and October 1 | 3 |
| Warm | October 20-22, 2009 | 6 |
| Cold | January 12-14, 2010 | 8 |
| Cold | March 2-4, 2010 | 2 |
| Cold | March 9-11, 2010 | 7 |
| Warm | July 6-8, 2010 | 2 |
| Warm | July 20-22, 2010 | 4 |
| Warm | September 14-16, 2010 | 1 |
| Cold | January 11-13, 2011 | 2 |
| Cold | February 15-17, 2011 | 1 |
| Cold | March 15-17, 2011 | 4 |


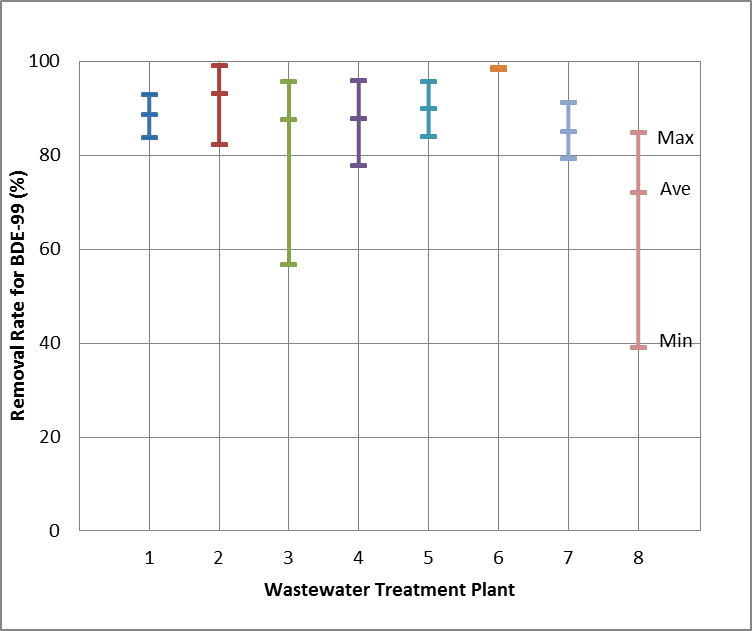


**Fig. S1 - Removal Rate for BDE-99 at Eight Wastewater Treatment Plants (The range of the removal rate is based on 12 data points for plants 1-2 and 6 data points for plants 3-8).**


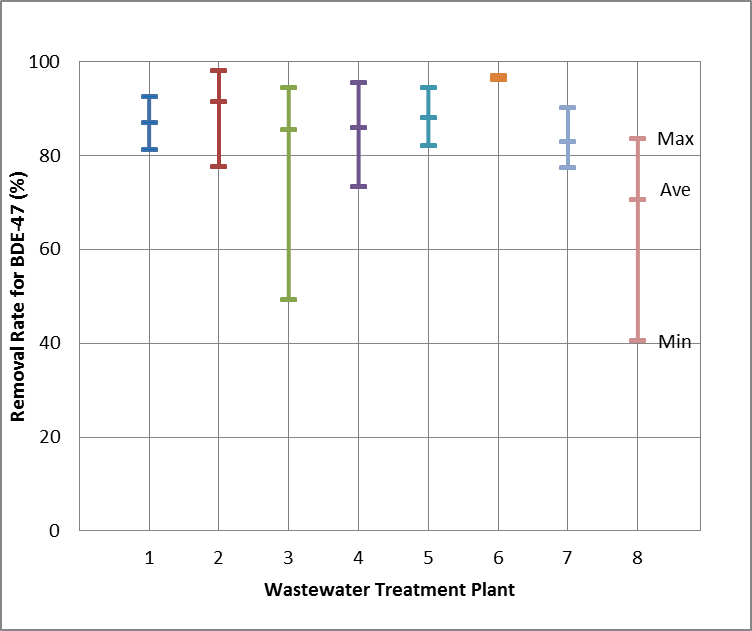


**Fig. S2 - Removal Rate for BDE-47 at Eight Wastewater Treatment Plants (The range of the removal rate is based on 12 data points for plants 1-2 and 6 data points for plants 3-8).**


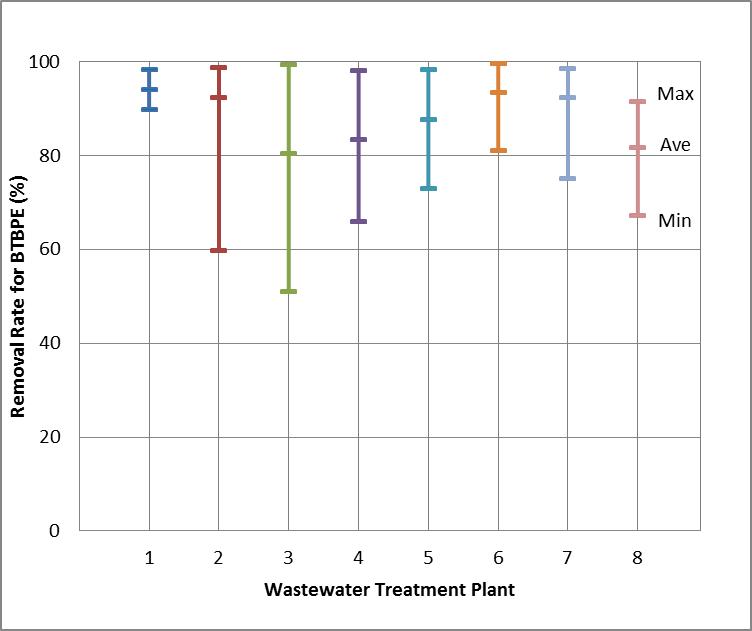


**Fig. S3 - Removal Rate for BTBPE at Eight Wastewater Treatment Plants (The range of the removal rate is based on 12 data points for plants 1-2 and 6 data points for plants 3-8).**


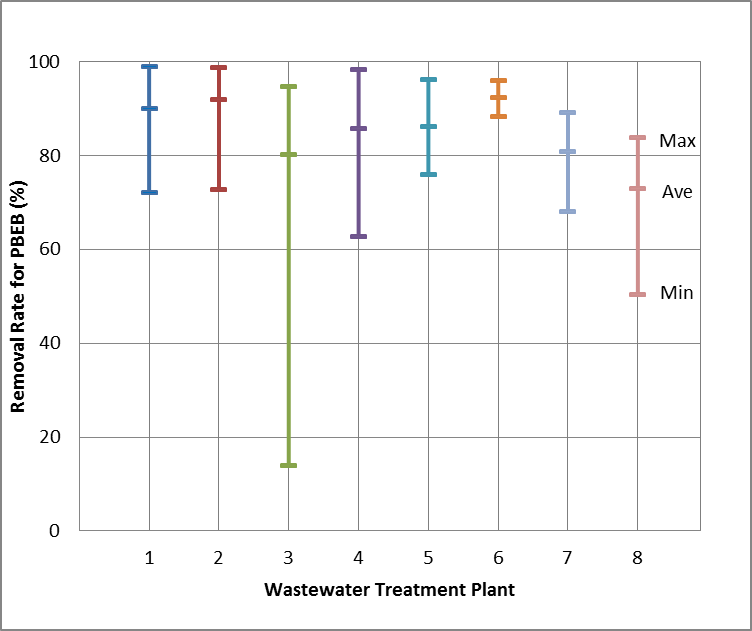


**Fig. S4 - Removal Rate for PBEB at Eight Wastewater Treatment Plants (The range of the removal rate is based on 12 data points for plants 1-2 and 6 data points for plants 3-8).**
